# Biopolymer-Based Microcarriers for Three-Dimensional Cell Culture and Engineered Tissue Formation

**DOI:** 10.3390/ijms21051895

**Published:** 2020-03-10

**Authors:** Lixia Huang, Ahmed M.E. Abdalla, Lin Xiao, Guang Yang

**Affiliations:** 1 Hubei Key Laboratory of Purification and Application of Plant Anti-Cancer Active Ingredients, School of Chemistry and Life Sciences, Hubei University of Education, Wuhan 430205, China; huanglixia@hue.edu.cn; 2 Department of Biomedical Engineering, College of Life Science and Technology, Huazhong University of Science and Technology, 1037 Luoyu Road, Wuhan 430074, China; ahmedbio1@hotmail.com

**Keywords:** biopolymer, polysaccharide, microcarrier, 3D cell culture, engineered tissue, bottom-up assembly

## Abstract

The concept of three-dimensional (3D) cell culture has been proposed to maintain cellular morphology and function as in vivo. Among different approaches for 3D cell culture, microcarrier technology provides a promising tool for cell adhesion, proliferation, and cellular interactions in 3D space mimicking the in vivo microenvironment. In particular, microcarriers based on biopolymers have been widely investigated because of their superior biocompatibility and biodegradability. Moreover, through bottom-up assembly, microcarriers have opened a bright door for fabricating engineered tissues, which is one of the cutting-edge topics in tissue engineering and regeneration medicine. This review takes an in-depth look into the recent advancements of microcarriers based on biopolymers—especially polysaccharides such as chitosan, chitin, cellulose, hyaluronic acid, alginate, and laminarin—for 3D cell culture and the fabrication of engineered tissues based on them. The current limitations and potential strategies were also discussed to shed some light on future directions.

## 1. Introduction

Traditional two-dimensional (2D) cell culture in plate has been found to distort cell behavior and biological functions to some extent as compared with cells in vivo [1,2]. As a typical example, 2D cultured tumor cells in vitro often show slower tumor progression and lower drug resistance in comparison to tumors in vivo, leading to a discounted efficiency of drug screening and testing [3,4]. This is mainly because 2D cell culture in vitro cannot establish efficient and multidirectional cell–cell and cell–extracellular matrix (ECM) interactions as present in the microenvironment in vivo, which potentially induce changes in cellular morphology and gene expression. To obtain cells with normal morphology, behavior, and functions as in vivo, the concept of three-dimensional (3D) cell culture was proposed, that is to culture cells in a 3D environment mimicking the in vivo microenvironment with sufficient and multidirectional cell-cell and cell-ECM interactions formed [5,6]. 

Different strategies have been proposed to achieve 3D cell culture, among which the scaffold-based approaches represent an important conceptual framework or paradigm [7]. The classic solid porous scaffolds created from biodegradable polymers have been extensively studied as a temporal template-like instructive support for cell attachment and growth. Efficient 3D cell attachment with excellent viability, motility, proliferation, and remodeling of the extracellular matrix can be achieved by using this approach due to the excellent biocompatibility and appropriate porous structure [8,9,10]. However, it is still technologically challenging to achieve the precise placement of different cell types within solid porous scaffolds and to achieve organ-like cell densities in tissue constructs [11]. A few reviews concerned with the advancement and shortcomings of the solid porous scaffolds are available [7,12,13,14]. Meanwhile, hydrogel-based scaffolds from natural and synthetic polymers have received broad attention for cell incubation due to their good biocompatibility, high water content, and special properties (such as stimuli responsiveness, injectability, etc.) [15,16,17]. A wide range of hydrogels have been developed as cell carriers for cell therapy and tissue engineering. Insight reviews on these topics can be found as well [18,19]. However, in terms of 3D cell culture, the difficulties in oxygen and nutrient supplement for inner cells in the hydrogels are becoming the bottleneck for the hydrogel approach [20,21].

Another classic approach for 3D cell culture is multicellular spheroids, formed without attachment to a scaffold [5,22]. Cell-cell adhesion is one of the key driving forces for the formation of the spheroids. By using this strategy, multicellular tumor spheroids have been reported and tested as in vitro tumor models for drug penetration and efficacy studies in tumors [23,24]. Compared to the 2D cultures in plates, tumor spheroids are more closed to the physiological tumor tissues in the main features, namely their structural organization, pH and nutrient gradients, hypoxia, protein expression, growth factor permeation, and interactions with ECM. The multicellular tumor spheroids support the interactions between tumor cells and normal cells. Thus, they may be used to study tumor progression by serving as models of tumor microregions and tumor growth at an early and avascular stage [25,26]. These spheroidal systems can also serve as equivalents of migrating micrometastases [25,27]. Besides, tumor spheroids are used to assess the release of soluble mediators, which provide cues for immunosurveillance and associated immune escape [28]. Overall, multicellular spheroids are acting as promising models to bridge the gap between traditional 2D in vitro studies and in vivo models [29]. Unfortunately, when the spheroids grow to a certain size, the transport of oxygen and nutrients to the core, and of metabolic waste products by simple diffusion becomes difficult, leading to a low concentration of oxygen and nutrients for the inner cells [5,30]. The spheroid diameter is thus limited to 200–400 μm due to these diffusion constraints. 

More recently, a cell accumulation technique was reported by Akashi et al. [31] for the rapid construction of 3D tissues. The authors first coated individual cells with fibronectin-gelatin (FN-G) nanofilms, which interacted with the α5β1 integrin receptors of cell membranes. The 3D tissues were formed by inducing cell-cell adhesion for the seeded cells in three dimensions. However, similar to multicellular spheroids, since the cells were tightly packed within the constructs without spacing, the transport of oxygen, nutrients, and metabolic waste to and from the inner cells became a major problem as the construct size increased [32].

Microcarrier-based approaches have been proposed for 3D cell culture using the micro-scaled scaffolds to support cell attachment and growth. They are considered to be more favorable for cell attachment with higher cell density and cell yields compared with the macroscopic scaffolds due to the higher surface area to mass ratio [33,34,35]. The transport of oxygen, nutrients, and metabolic waste within the microcarriers can also be easier in comparison with cellular spheroids when a porous structure is applied. In view of these advantages, the microcarrier technique has been widely studied over the past few decades for the purpose of 3D cell culture, with major applications in cell therapy and tissue repairing [36,37,38,39,40,41,42,43]. In recent years, the microcarrier-based approach of 3D cell culture has also opened a door for fabrication of engineered tissues, which is one of the cutting-edge topics in tissue engineering and regeneration medicine. Different engineered/artificial tissues have been developed from cell-laden microcarriers either as model tissues for pharmacological and pathological studies or for tissue regeneration [44,45,46,47].

Several reviews on the microcarrier technology are available, discussing its advantages in cell expansion and applications in tissue engineering [48,49,50,51,52]. As an example, Li et al. [51] overviewed the microcarrier culture technology with emphasis on the microcarrier materials, classification, and applications as cell-delivery systems for tissue repairing. More recent advances in the use of microcarriers for cell cultures were reviewed by Chen et al. [52]. The authors also discussed the significance of microcarriers as an ex vivo research tool and their therapeutic potential in vivo. However, the concepts of 3D cell culture using microcarriers and in vitro tissue formation through engineering cell-laden microcarriers as building blocks have not been fully addressed. In this review, we will first focus on the concept of 3D cell culture using microcarriers as support. The microcarriers discussed in this work include not only microspheres but also microgels with materials limited to biopolymers, such as chitosan, cellulose, hyaluronic acid, alginate, and poly(D,L-lactide-co-glycolide) (PLGA). Then, the fabrication of engineered tissues using cell-laden microcarriers as building blocks through a bottom-up assembly strategy and their benefits in biological and medical studies will be discussed. An in-depth look into the advancements and limitations in the field and a general prospective will be presented as well. We hope this review can provide comprehensive information and inspire new thoughts in the microcarrier concerned areas with purposes of 3D cell culture and engineered tissue formation.

## 2. Fabrication Techniques for Three-Dimensional (3D) Cell Microcarriers

An emulsification-based process is frequently employed to fabricate microspheres from different polymers such as cellulose, chitosan, and PLGA. Combining the emulsification method and biomimetic mineralization process, Patricio et al. [53] prepared superparamagnetic microspheres made of collagen type I-like peptide matrix mineralized with Fe^2+^/Fe^3+^ doping hydroxyapatite. The microspheres were obtained by emulsifying the mixed slurry in the presence of citrate ions. The achieved biomimetic surface showed enhanced bioactivity and dispersion ability in the cell medium [53]. By using a process combining micro-emulsification and thermally induced phase separation (TIPS), we prepared chitosan microspheres with a highly porous structure as microcarriers for 3D cell culture [54]. Besides, the water-in-oil (W/O) emulsification process was combined with the freeze-drying process to prepare aerogel microspheres from the hybrid of poly(vinyl alcohol) (PVA) and cellulose nanofibril (CNF) [55]; while the combination of the oil-in-water (O/W) single-emulsion method and porogen leaching-phase separation process was applied to prepare PLGA porous microspheres with a size of approximately 50 µm for cell microcarrier application [56]. However, the emulsification-based method has limitations on the material range. As an example, microspheres cannot be obtained from the nanofibers of bacterial cellulose (BC) by this process without dissolving the nanofibers. Recently, Higashi et al. [57] reported the successful preparation of nanofibrous microspheres of BC through a template-assisted microbial fermentation approach.

**Figure 1:**
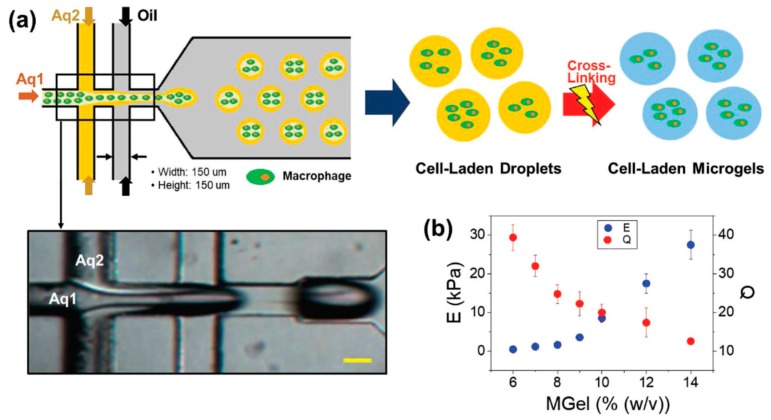
Schematic illustration of a double flow-focusing microfluidic fabrication of macrophage-laden microgels (**a**). The image below shows the delineated core (Aq1) and shell (Aq2) flows during droplet generation (scale bar: 100 μm). (**b**) Elastic moduli (E) and swelling ratios (Q) of photo-crosslinked MGel hydrogels at various concentrations (mean ± SD, *n* = 6) [65]. (Reproduced with permission from Wiley.)

Microfluidics is a versatile method to obtain intricate microparticles with varying size and shape by producing emulsion droplets in microscale [58,59,60,61]. Monodisperse droplets can be efficiently developed and further engineered to generate microgels, which provides a valuable platform for 3D cell culture and miniaturized tissue engineering [62,63,64,65,66]. Lee et al. [65] fabricated macrophage-laden microgels containing methacrylic gelatin for microtissue fabrication using a double flow-focusing microfluidic approach. Briefly, monodisperse droplets of gel precursor solution infused with cells were first generated by a microfluidic device having a flow-focusing geometry, followed by photo-crosslinking to generate cell-laden microgels (Figure 1). Similarly, methacrylate laminarin microparticles were also prepared by combination of microfluidics technology and photopolymerization [67]. Microfluidic-based cell encapsulation by microgels has been applied in studying cellular dynamics and interactions. Pajoumshariati et al. [68] encapsulated crypt and Peyer’s patch cells separately into distinct alginate/gelatin microgels using the microfluidic approach. The resultant cell-laden microgels allowed for intercellular chemical communication, and could be used as a model system for investigation of cell–cell interaction.

Different from the current microfluidic-based cell microencapsulation techniques in which fluorinated oils are primarily used, the authors investigated the use of different nonfluorinated oils as the carrier phase in cell encapsulation. Liu et al. [69] developed a high-throughput double emulsion-based microfluidic approach to produce poly(ethylene glycol) (PEG)-based microspheres with chemical functionality. The microspheres containing primary amines (chitosan, CS) or carboxylates (acrylic acid, AA) were prepared through formation of double emulsion droplets by high-throughput capillary microfluidic approach. 

With the assistance of droplet microfluidics, multicomponent reactions, typically the Passerini three-component (P-3CR), and the Ugi four-component reaction (U-4CR), can be applied to produce microgels with a rather uniform size [70,71]. Unlike the regular methods of microgel formation, where the hydrogel building blocks need to be premodified to introduce certain functions, multicomponent reactions can simply introduce and extend functions by changing one block to another in a library-like fashion [72,73]. As an example, Hauck et al. [70] reported the single-step synthesis of micro-sized polysaccharide based multifunctional gels through multicomponent reactions, in particular P-3CR and U-4CR. The synthesis of functional polysaccharide one-step was reported by selecting polysaccharide as substrate, selecting homogenous multiple (ethylene glycol) as crosslinking agent component, taking the third component of heterogeneous function as a functional agent, and isocyanate as the starting component of the reaction. These materials were processed into non-colloidal gels using droplet-based microfluidic, and their size distribution was as low as 1% to 2% [74].

Other approaches for fabricating microcarriers were also reported. For example, Zhang et al. [75] proposed an acid-dissolution/alkali-precipitation process to prepare chitosan-based microcarriers, which were reinforced with graphene oxide. Table 1 summarizes the current techniques for fabricating 3D cell microcarriers including microspheres and microgels.

## 3. Microspheres as 3D Cell Carriers

Microcarrier culture technology was first proposed by van Wezel in 1967 [35]. In this technique, porous or non-porous microspheres prepared from various materials are employed as supports for anchoring cell lines. It has shown great potential to culture a variety of animal cells in high yield, due to the advantages such as high specific surface area, suspension culture with homogeneous stirring and easy scale-up [33,34]. Different microcarriers are commercially available which are manufactured from dextran (Cytodex), cellulose (Cytopore), gelatin (Cultispher), polystyrene (HLXⅡ-170), polyethylene and silica (Cytoline), and glass (G2767) [76,77]. Unfortunately, the commercially available microcarriers have limitations in offering tailored properties such as elastic modulus and special biological cues. Correspondingly, extensive efforts are being made to develop microcarriers with specific considerations of structure and function. A summary of current microspheres including non-porous and porous microspheres and their applications is shown in Table 2.

### 3.1. Non-Porous Microspheres

Collagen type I-like peptide matrix was first mineralized with Fe^2+^/Fe^3+^ doping hydroxyapatite, and then used to prepare microspheres with superparamagnetic properties for osteoblast cells culture. [53]. Osteoblast cells maintained their typical morphology without obvious damage, whereas showing up-regulation of osteogenesis markers. The cellular activity and bio-resorption ability of these cells could be triggered in response to the release of calcium and iron ions from the microspheres in culture media that simulate physiological or inflammatory environments.

Chitosan-based microspheres were also applied as microcarriers for cell culture [75,78,79,80], because of the non-toxicity and good biodegradability of chitosan [81,82]. Zhang et al. [75] developed hybrid microspheres from chitosan and graphene oxide, which supported stem cell expansion, growth, and proliferation. Custódio et al. [78] reported monoclonal antibodies-conjugated chitosan microspheres. Cells were first captured by the monoclonal antibodies and subsequently grew and expanded on the microspheres.

Multifunctional methacrylate laminarin microparticles were fabricated to support cell attachment and expansion. Platelet lysates were loaded in the microparticles and an adhesive peptide was further conjugated on the surface to improve cell adhesiveness and expansion. Moreover, microparticles aggregation linked by the expanded cells was observed to form robust 3D constructs, which suggested that these methacrylate laminarin microparticles could provide a platform for rapid fabrication of large tissue engineered constructs through assembly of cell-laden microparticles by the action of cells [67]. Duan et al. [39] constructed chitin microspheres with a nanofibrous architecture using a bottom-up approach. The chitin microspheres showed a large specific surface area and a homogeneous nanofibrous structure throughout, which was favorable for application as microcarriers. The chitin microspheres were seeded with human hepatocyte L02 and the results indicated cells could adhere to the chitin microspheres with a high attachment efficiency. The results of cell attachment indicated that the chitin microspheres may serve as promising microcarriers for 3D cell culture and tissue engineering applications.

Besides the natural biopolymers, some synthetic biopolymers were also prepared into microspheres as microcarriers. Microspheres from polylactide (PLA) with controlled biochemical cues represent promising synthetic microcarriers for cell culture and injectable platform of cell treatment. For example, PLA microspheres coated with collagen were used for in vitro chondrocyte culture. They provided effective supports for cell attachment, proliferation, and spreading, which were in particular enhanced by higher content of collagen [83]. Another study reported RGD (Arg-Gly-Asp) peptide modified PLA microspheres as microcarriers, which were applied to generate tissue engineering cartilage in a flow intermittency bioreactor [84]. It was observed that 3D aggregates were formed from the cell-seeded microspheres after seven days of culture, which gradually grew over 14 days. The growth rate of the 3D aggregates was positively correlated to the content of RGD. These 3D aggregates as building blocks may be used for nascent tissue formation in high-porosity thick scaffolds or in hydrogels.

### 3.2. Porous Microspheres

Zhang et al. [55] reported highly porous aerogel microspheres with a low bulk density of 0.0047 g/cm^3^ from crosslinked hybrid of poly(vinyl alcohol) (PVA) and cellulose nanofibril (CNF). The pore size of microspheres varies from nanometers to micrometers. The highly cross-linked aerogel microspheres have strong robustness and can maintain their shape in cell culture. Cell seeding, attachment, and proliferation on the surface of these aerogel microspheres were verified by living/dead assay. Some cell migration into the internal pores of the structure was observed by fluorescence images. Moreover, cell behaviors such as adhesion, differentiation, and proliferation were promoted effectively by the interconnected and porous nanofiber structure of microspheres. 

There are many studies focusing on PLGA porous microspheres as carriers for cell expansion, such as pluripotent stem cells [85]. The porous structure of PLGA microspheres can not only act as a scaffold supporting the stem cell growth, but can also serve as a delivery system for bioactive factors, which are used to modulate cell differentiation. The porous PLGA microspheres also support the formation of spherical cellular aggregates when used as microcarriers by suspension cultivation. For this purpose, Chung et al. reported PLGA porous microspheres with a size of 50 µm, which were prepared through combining the porogen leaching phase separation process with the oil-in-water single-emulsion method [56]. Due to the appropriate density and porous structure, the PLGA microspheres could be used as effective microcarriers for suspension culture. It was observed that by suspension cultivation in a spinner flask, these porous microcarriers seeded with 3T3 L1 mouse preadipocyte cells supported the formation of spherical cellular aggregates, which could be potentially used for soft tissue augmentation and reconstruction. Kim et al. [86] fabricated porous PLGA microspheres using effervescent salt, ammonium bicarbonate as pore-foaming agent. Highly open PLGA microspheres were used as suspension microcarriers for culturing NIH 3T3 cells in a spinner flask. After seven days of cultivation, the spherical cells were observed to be present within the microsphere pores in large quantities. This PLGA microcarrier system could be used as injectable and biodegradable scaffold. 

Porous polyelectrolyte complex (PEC) microspheres were prepared from poly(L-glutamic acid) and chitosan through electrostatic interaction [40]. The porous PEC microspheres supported chondrocyte attachment and proliferation more efficiently than the chitosan microspheres. Compared with the chitosan microspheres, significant more cartilaginous matrix was produced by the chondrocyte-seeded PEC microspheres after injection subcutaneously for eight weeks. These results indicated that the porous PEC microspheres had the high potential to be used as an injectable platform for cartilage repair and regeneration. Porous microcarriers can also be prepared from decellularized tissues. As an example, decellularized adipose tissue was employed to fabricate microcarriers for dynamic culture and expansion of human adipose-derived stem/stromal cells [42].

## 4. Microgels as 3D Cell Carriers

Microgels provide a 3D environment mimicking the ECM characteristic of high-water content, which allows for cell migration and nutrient/waste exchange [87,88]. Superior than the microspheres, encapsulated cells within microgels can be protected from damage by shear force during injection. Moreover, compared with bulk hydrogels, microgels allow more efficient nutrient/waste exchange due to the micro-scaled size. Microgels based on natural biopolymers provide a promising platform for cell culture and tissue engineering due to their superior biocompatibility and high degree of flexibility in imparting a range of functions.

Gelatin-based microgels are widely applied to encapsulate cells and support cell growth and function for different purposes. As an example, Sung et al. [89] developed gelatin-based microgel spheres for the encapsulation of mesenchymal stem cells (MSCs) with a tunable mechanical stiffness through controlling the crosslinking degree of gelatin matrix by genipin. It was shown that these gelatin microgels supported the viability and differentiation of the MSCs in a pro-inflammatory environment. Moreover, it was found that the morphology of MSCs was affected by the stiffness of gelatin matrix. The cells showed a more spread morphology when the microgels were softer, while cells with a more elongated morphology were observed when stiffer matrix was used for the microgels. Photo-crosslinked microgels with varying mechanical properties were also obtained from methacrylic gelatin for use as a 3D cell culture platform [65].

Microgels provide a promising platform for studying cell-cell interactions and fabricating spheroids and organoids. Pajoumshariati et al. [68] reported microfluidic-based alginate/gelatin microgels for cell encapsulation and cellular interactions. The gelatin, containing the cell adhesive amino acid motifs, could improve cell adhesion and proliferation. Moreover, the biodegradability of gelatin could further support the formation of healthy organoids [90]. Lee et al. [65] fabricated bioactive spherical microgels supporting 3D cell culture to create tumor spheroids. Uniform-sized aqueous droplets of methacrylic gelatin solution dispersed with breast adenocarcinoma cells, MCF-7, were generated by a flow-focusing microfluidic device and subjected to photo crosslink to fabricate cell-laden microgels. It was found that the mechanical properties of the microgels, controlled by the concentration of methacrylic gelatin, had an impact on cell proliferation and the eventual spheroid formation. Moreover, the role of macrophages of fibroblasts on tumoral spheroid formation within the microgels was explored as they are known to play a prominent role in tumor physiology.

Besides gelatin, there are many other natural biopolymers having been used to fabricate microgels for 3D cell culture. Polysaccharides such as hyaluronic acid, alginate, and chitosan have been used for microgel formation and have broad applications in cell cultures and tissue engineering because of their suitable properties of non-toxicity, biocompatibility, non-immunogenicity, and biodegradability [66]. For example, Hauck et al. [70] reported the synthesis of multiple microgels from hyaluronic acid, alginate, and chitosan via P-3CR and U-4CR.

**Figure 2:**
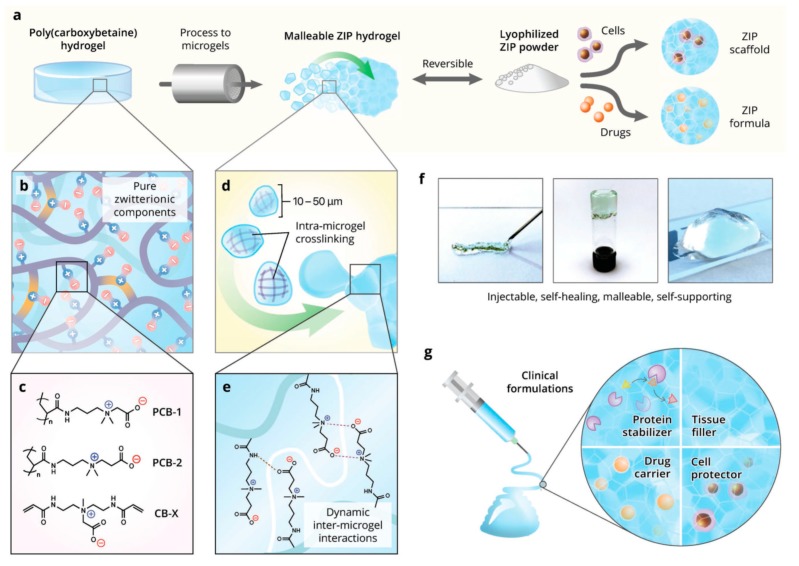
Production, properties, and applications of zwitterionic injectable pellet (ZIP) hydrogels. (**a**) Overview schematic showing the production of viscoelastic ZIP gels, which can be lyophilized for simple formulation and mixed with cells or therapeutics. (**b**) Hydrogel components are purely zwitterionic, consisting of (**c**) carboxybetaine acrylamide polymers (PCB-1 or PCB-2) with carboxybetaine diacrylamide crosslinker (CB-X). (**d**) Covalent crosslinks inside each microgel enable bulk support and elasticity. (**e**) Dynamic zwitterionic fusion interactions enable reconstruction of microgels into new viscoelastic ZIP material. (**f**) ZIP gels can be injected through needles, self-heal, and retain their shape. (**g**) Applications of ZIP gels include injectable soft tissue fillers, therapeutic carriers, and cell scaffolds for growth and protected injection [99]. (Reproduced with permission from Wiley.)

Among the large amount of natural and synthetic polymers that can be used to prepare hydrogels, the polymers containing balanced pairs of cationic and anionic groups, namely polyzwitterions, have received special attention for their unique biocompatibility [91,92,93]. Due to the zwitterionic feature, these polymers are able to mimic phospholipids in cell membranes [94] or mixed-charged surfaces of proteins [95]. Zwitterionic polycarboxybetaine (PCB) was used to prepare hydrogels for applications in cell encapsulation with remarkable biocompatibility, physiological stability, and nonimmunogenicity [96,97,98]. However, generation of free radicals by photopolymerization of carboxybetaine acrylamide monomers to form PCB hydrogels is harmful to the encapsulated cells. Recently Sinclair et al. [99] reported self-healing zwitterionic microgels through extruding the bulk PCB hydrogels through micronic steel mesh. The microgels can be easily tuned to any microscale size by changing the mesh pore size. Through a dual mechanism of covalent crosslinking within microgels and supramolecular interactions between the microgels, the zwitterionic microgels can be rebuilt to constructs of zwitterionic injectable pellet (ZIP) with favorable moduli and tunable viscoelasticity (Figure 2). It is notable that the powder obtained by lyophilizing the ZIP constructs can be fully recovered by rehydration in terms of strength and elasticity. Moreover, injectable or malleable PCB/cell or PCB/drug hydrogels can be obtained by reconstituting the lyophilized powder with cell or therapeutics suspensions, where the mixtures self-heal into a homogeneous composite construct rapidly and spontaneously (Figure 2).

Microgels provide a biomimetic microenvironment mimicking natural ECM, the properties of which play a vital role in cell adhesion, cell interactions, and spheroid formation. It was suggested that the surface wettability, chemistry, and charge of the microgels influenced cell attachment, proliferation, and migration, which are essential for the formation of multicellular spheroids. Furthermore, the formation progress of tumor spheroids and their size distribution are greatly affected by the surface charge density and hydrophobicity of the microgel microenvironment. Cui et al. [100] demonstrated the quick formation of large spheroids using microgels with a moderate negative charge density and a highly hydrophilic surface, which promoted cell proliferation. Different microgels and their application as cell carriers are summarized in the following Table 3.

## 5. Understanding of Real 3D Cell Culture and Challenges

In terms of 3D cell culture, it is true that many types of microcarriers have been reported; however, for most existing carriers, cells can only attach and grow on the outermost surface or the external pore surface, which limits the formation of multidirectional and sufficient cell–cell interaction. Consequently, these cannot be considered “real” 3D cell cultures. We refer the readers to the definition of “real” 3D cell culture as defined in the literature [5], namely a system able to mimic the in vivo environment that cells experience in native organs and tissues, where cells grow in a 3D space rather than on a surface, with multidirectional cell-cell interactions. This is crucial for the cells to present normal morphology and functions, as they are found in vivo [5]. It should be pointed out that “real” 3D cell culture with microcarriers is very different from that achieved with macroscopic scaffolds which have been reported previously by several groups [101,102], while microcarrier culture is still very challenging. This is mainly because the fabrication of microspheres having a porous structure with dimensions appropriate to allow multidirectional cell-cell interactions and sufficient mechanical strength is much more difficult than for macroscopic scaffolds. To make the microcarriers sufficiently rigid, the preparation of biopolymer-based microcarriers often involves chemical crosslinkers such as glutaraldehyde. These microcarriers inevitably contain and release toxic crosslinking agents during the cell culture process and is infaust to cell growth. Therefore, developing safe and high-performance microcarriers for “real” 3D cell culture is both desirable and crucial, and represents an important development in the microcarrier field. 

Porous microcarriers were fabricated from the nanocomposites of strontium-substituted hydroxyapatite-graft-poly (γ-benzyl L-glutamate) [103]. It was confirmed by confocal microscopy that the seeded rabbit adipose-derived stem cells (ADSCs) adhered and infiltrated within the internal pores of the microspheres. Further studies demonstrated that ADSCs appeared at different depths (i.e., 1/8, 2/8, 3/8, and 4/8 diameter) of the microcarriers, which suggested that cells could attach and grow in the innermost areas of the microcarriers. Three-dimensional cell cultures by natural biopolymer-based microcarriers were also achieved although few examples were available. Fang et al. [40] reported poly(L-glutamic acid)-coated porous chitosan microcarriers for the 3D culture of chondrocytes, enabling cell migration to the inside of the carrier and cellular multidirectional contacts. The 3D cell culture within microcarriers was clearly demonstrated for the first time. The authors confirmed that high-performance 3D cell culture with these microcarriers was mainly enabled by the presence of the poly(L-glutamic acid) coating, which increased the hydrophilicity of the microcarriers. Their results indicated that the unmodified chitosan microcarriers did not work so well, and no results were provided to support the 3D chondrocyte culture with the unmodified chitosan microcarriers. However, we recently demonstrated that chitosan microcarriers without surface modification can also support high-performance 3D cell culture. Highly porous chitosan microspheres (CSM) were prepared using an emulsion-based thermally induced phase separation (TIPS) method and evaluated as microcarriers for 3D culture of hepatocyte cells. Due to their good biocompatibility and unique porous structure, hepatocytes can not only adhere to the surface of microspheres, but also grow into the internal pores, and form multidirectional cell-cell connections [54]. It was then disclosed that the interconnected porous structure of the microcarriers with pores of 20 to 50 µm is crucial for the successful 3D cell culture, in addition to the good biological properties of chitosan. Previous studies have shown that the porous structure plays a vital role in cell culture with microcarrier systems, because it not only promotes cell adhesion, migration, and distribution, but also promotes the exchange of nutrients and metabolic waste [104]. In addition to the high porosity, a suitable pore size range is also key to achieving high-performance cell culture using porous microcarriers or scaffolds [105,106,107]. If the pore size is too small or too large, it may lead to restricted cell migration and adhesion, respectively [105]. Finally, pore interoperability is also important, especially for forming sufficient and multidirectional cell-cell connections. 

## 6. Microcarrier-Supported 3D Cell Cultures towards Engineered Tissues

Engineered tissues have broad application prospects in the fields of tissue models for drug screening and evaluation, disease development and control, tissue engineering, and regenerative medicine. In recent years, it has been found that because of the different microenvironment of cells, the cell lines of 2D amplification in vitro are significantly different from those of natural tissues in phenotype and genotype, which limits their application in related fields [1,2]. While on the other hand there are problems with natural tissues, such as shortage of sources, species and individual differences, and social ethics. This situation inspires the researchers to simulate and replace natural tissues by building engineered tissues. 

Through a bottom-up tissue engineering strategy, cell-laden microcarriers have great potential to be used as modular building blocks to recreate complex tissues with hierarchical and biologically functional structures. The cell-laden microcarrier building blocks can be controllably assembled into larger tissue constructs via specific interactions such as click chemistry, enzymatic crosslinking, covalent crosslinking and magnetic interactions, or randomly assembled via simple mixing, accumulation, or stacking.

### 6.1. Engineered Tissues from Controllable Assembly via Specific Interactions

Lee et al. [65] reported a 3D hydrogel-based co-culture tissue model based on cell-laden microgels. Microgels encapsulating macrophage were first prepared by photo-crosslinking cell-laden droplets containing methacrylic gelatin, which were produced by using a microfluidic flow-focusing device. Macrophage microtissues were formed from these cell-laden microgels. After that, co-culture tissue models were developed by transferring the macrophage microtissues into large tissue constructs containing either normal or cancer cells. By using these co-culture tissue models, the effect of mutual influence between two different cell types was systematically examined. 

Caldwell et al. [108] prepared microgels with clickable surface groups from PEG end functionalized with azide and alkyne moieties. The synthesized microgels with average diameters either in the order of 100 or 10 µm were then introduced to a cell suspension. Cell-laden macroscopic and microporous hydrogels were formed via clicking the microgel blocks. To obtain a more biomimic tissue construct, a fibronectin-derived adhesive peptide, Gly-Arg-Gly-Asp-Ser (GRGDS), was introduced into the microsphere formulation to promote cell spreading, cell-particle interactions, and cell-cell interactions. Segura et al. proposed to assemble cell-laden microgels using enzymatic crosslinking for wound healing and brain regeneration [109,110]. 

**Figure 3:**
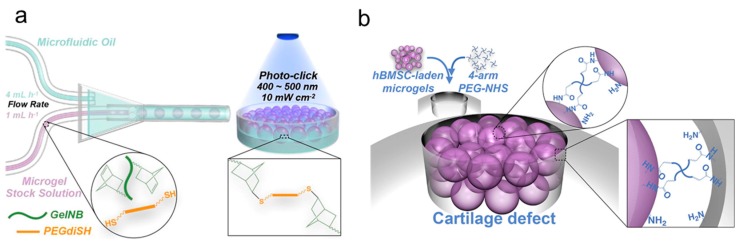
Scheme illustrating microgel formation using a pipette tip-based microfluidic device (**a**). The 4-arm poly(ethylene glycol)-N-hydroxysuccinimide (PEG-NHS) was used as covalent crosslinker for bottom-up assembly of human bone marrow derived mesenchymal stem cells (hBMSC)-laden microgels for tissue formation (**b**) [111]. (Reproduced with permission from Elsevier.)

Frith and coworkers [111] designed tissue-like structures for cartilage tissue formation through bottom-up assembly using cell-laden microgels as the building blocks. Microgels of gelatin norbornene (GelNB) containing human bone marrow derived mesenchymal stem cells (hBMSC) were first produced with a microfluidic device and then cured under visible light (Figure 3a). The assembly of hBMSC-laden microgels was achieved by covalent crosslinking using 4-arm poly(ethylene glycol)-N-hydroxysuccinimide (PEG-NHS) as the crosslinker, which also induced covalent bonding between the microgels and the surrounding tissue construct (Figure 3b). It was found that the resultant tissue mimetic constructs could provide physical and biological cues for chondrogenesis of hBMSC, which promoted the formation of hyaline cartilage structure. 

Demirci et al. [112] reported a method of magnetic assembling microgel modules encapsulating cells to fabricate 3D anisotropy artificial tissues. They first prepared the microgel modules of different shapes and wrapped the same or different cells in the modules, and then immersed the microgel modules into the free radical solution to make them magnetically responsive. These cell-laden microgel modules can be assembled into 3D heterogeneous composite structures under the drive of the applied magnetic field. This method can realize controllable assembly of different kinds of cells and has potential in constructing the complex 3D anisotropic tissues.

### 6.2. Engineered Tissues from Random Assembly of Cell-Laden Blocks

**Figure 4:**
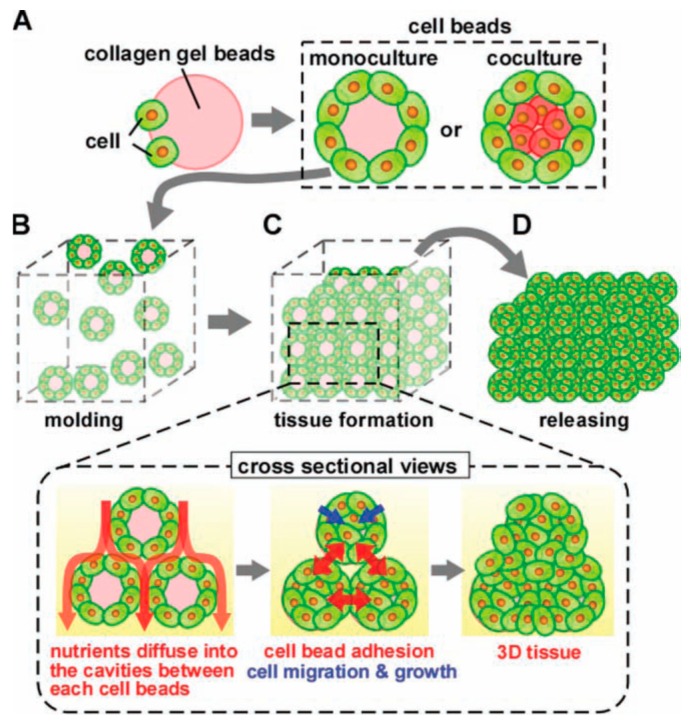
Concept of bead-based tissue engineering: Monodisperse cell beads are molded into a 3D tissue architecture [113]. (Reproduced with permission from Wiley.)

Besides the specific chemical and physical interactions used as driving forces to assemble building blocks into artificial tissues, random assembly like simple mixing, dispersion, accumulation, or stacking was also widely applied to fabricate artificial tissues from cell-laden building blocks in microscale [113,114,115,116,117,118]. Fan et al. [118] fabricated microgel modules similar to bone units by loading the human osteoblasts MG63 and collagenase into the collagen microspheres, the surfaces of which were then inoculated with human umbilical vein endothelial cells (HUVEC). Then the modules were randomly assembled with gelatin microspheres. With the degradation of gelatin microspheres, vascular microtubule structures were gradually formed by HUVEC cells, resulting in a 3D engineered tissue with cell arrangement and inner connected tubes. Matsunaga et al. [113] reported the rapid construction of macroscopic 3D tissue constructs using a method of molding cell beads as shown in Figure 4. Firstly, cells were cultured over the surface of collagen microgels, which may also encapsulate cells of another type. The consequent constructs, namely cell beads, were then used to form 3D tissues by random stacking into a prefabricated silicone mold. The cavities between the cell beads allowed the medium to be diffused into the 3D architecture, thus supplying nutrition for the cells. Cell-cell connections were formed between cells located on the surface of the beads. Cells migrated and grew within the collagen microgel beads and finally formed the 3D tissues.

Different from the existing 3D constructs based on assembly of cell-laden microgels, Wang et al. [115] proposed hydrogel-encapsulating cell-laden microcarriers for tissue engineering purposes such as bone regeneration. The cell-laden microcarriers were based on gelatin-grafted-gellan (TriG) microspheres on the surface of which anchorage dependent cells (ADCs) such as osteoblasts were attached. Then the cell-laden microcarriers were suspended in liquefied low gelling-point agarose gel to form the construct of hydrogel-encapsulating cell-laden microcarriers. Compared with the macroscopic cell-laden agarose gel, microcarriers acting as anchors played an important role in the cell destiny. They not only provided robust and sufficient support for cellular adhesion, but also promoted the cells to spread out into their native morphology. The composites showed a competent potential in application as injectable vehicle for the conveyance of ADCs and regenerations of bone and other tissues. Cell-laden microgels or microspheres for fabricating 3D engineered tissue are summarized in Table 4.

### 6.3. Advantages, Limitations and Prospective

As mentioned in the Introduction, there are different strategies proposed to achieve 3D cell culture and subsequent engineered tissues, such as the macroscopic scaffold-based approaches. Compared with these methods, the microcarrier-based approach is more favorable for cell attachment with higher cell density and yields because of higher surface area to mass ratio than the macroscopic scaffolds, in which the transport of oxygen, nutrients, and metabolic waste within the microscaffolds is much easier compared to cellular spheroids and bulk hydrogels, because of the porous structure or smaller size of the microcarriers. With respect of engineered tissue fabrication, engineered tissues can be obtained from cell-laden 3D microcarriers as building blocks through a bottom-up assembly strategy. This method has potential comprehensive advantages in the construction of multi-cell species, high cell activity, and cell ordered arrangement towards engineered tissue formation. In particular, during the early stage of assembly, the gaps between modules were conducive to the diffusion of culture medium, providing a way of thinking for the nutritional supply of cells in addition to vascularization in the constructs. However, with the growth and proliferation of cells, the gaps between modules will quickly disappear, still resulting in a lack of supplement in the constructs. To overcome this bottleneck of supplement, one of the promising ways is vascularization in the constructs by co-culturing HUVEC cells, although there are still challenges in this area. Recently, we proposed a supramolecular way to solve this problem. Briefly, cell-laden microcarriers containing host domains or guest domains were prepared. Dynamic and reversible assembly was then achieved using these cell-laden microcarriers as building blocks via host-guest interactions. This supramolecular system exhibited reversible sol-gel transition responsive to light switches or competitive host or guest species. The breathing-like property allowed culture medium to draw in and release during the cell proliferation and tissue formation.

Finally, there is a considerable difference to using microspheres and microgels as microcarriers in terms of 3D cell culture and tissue engineering. Microspheres, as solid scaffolds, usually possess high compression moduli and large surface areas, which are favorable for cell adhesion and spreading. Moreover, a well-fabricated porous structure may allow sufficient cell migration and maximum cell-cell interactions [45]. Microgels are usually used as microcarriers through cell-encapsulation strategies. They provide an ECM-like microenvironment with high water content and good biocompatibility, which promote cell survival and spheroid formation in the short term. However, the highly hydrophilic microenvironments may also constrain the suspended cells to a round shape, which decreases the cellular activity and function. Moreover, the surrounding hydrogel matrix may impede cell spread and migration, resulting in fewer cell interactions [45]. Based on these features, microspheres are relatively more widely applied for purpose of large-scale cell expansion, while microgels are more suitable as injectable platforms for cell delivery and therapy.

## 7. Conclusions

Biopolymers, mainly including natural polysaccharides, proteins, and a few biodegradable synthetic polymers, have been used to develop 3D microcarriers including porous and non-porous microspheres and microgels, which provide a versatile platform for cell culture and microtissue formation. Cells are attached to the microcarrier surface or grow into the internal area to form a cell/material composite construct. It is worth noting that, in a strict sense, the real 3D cell culture should not only allow cell attachment and growth on outermost surface of the microcarriers, but also enable cell growth within their internal structures, with sufficient and multidirectional cell-cell interactions. This is important for the cultured cells to maintain normal morphology and function as in vivo. Therefore, microcarriers with an optimized porous structure and appropriate pore size besides good biocompatibility are desired in future studies. The 3D cell cultures with the microcarriers can be further processed to larger tissue constructs through controllable or random assembly. Different engineered tissues have been developed and preliminarily used in biological and medical studies, including in vitro studies as platforms for drug evaluation and disease models, and in vivo applications for tissue repair and regeneration. However, challenges still remain in fabrication of complex functional tissues especially in nutrition supplement aspects. Vascularization in the constructs and other possible approaches such as supramolecular assembly may be further studied to overcome this issue.

## Figures and Tables

**Table 1:** Technology for constructing three-dimensional (3D) cell microcarriers.

Morphology	Method	Size (µm)	Ref.
Non-porous microsphere	Combining the emulsification method and biomimetic mineralization process	70	[53]
	Fermentation by specific bacteria	200–1000	[57]
	High-throughput double emulsion-based microfluidic approach	100	[69]
	Through an acid dissolution/alkali precipitation approach.	400	[75]
Porous microsphere	Micro-emulsification and thermally induced phase separation (TIPS)	150	[54]
	Combination of the water-in-oil (W/O) emulsification process and the freeze-drying process	100–500	[55]
Microgel	Using a microfluidic flow-focusing device	100–160	[65]
	Combination of microfluidics technology and photopolymerization	100	[67]
	Microfluidic approach	50	[68]
	Multicomponent reactions	40–80	[70]
	Droplet based microfluidic	Micro-size	[74]

**Table 2:** Microspheres and their applications in cell culture.

Microspheres	Materials	Size	Cell Type	Application	Ref.
Non-porous microsphere	Collagen	70	Osteoblast cells	Potential ability of drug carrier and smart response in the presence of inflammatory states.	[53]
	Chitosan	400	Human umbilical cord mesenchymal stem cells (huMSC)	Support long-time stem cell expansion can greatly maintain the pluripotency of huMSC	[75]
	Chitosan	115	Human subcutaneous adipose cell	separation, scale-up expansion of specific cell type and successful use as an injectable system to form small tissue constructs in situ.	[78]
	Chitin	3–130	Human hepatocyte L02	As excellent 3D cell carriers for applications in tissue engineering.	[36]
	Polystyrenene (PS)/ Poly(ethylene glycol) (PEG)	400–500	Human mesenchymal stem cells (hMSC)	These microcarriers with defined, synthetic coatings may be suitable for a variety of bio-manufacturing applications.	[38]
	Polylactide (PLA)	180–200	Chondrocyte	Collagen-coated PLA microspheres could effectively support the attachment and proliferation of chondrocytes.	[83]
	Poly-L-lactide (PLLA)	100–200	Human chondrosarcoma line OUMS-27	Used as building blocks for developing nascent tissue for clinical use.	[84]
Porous microsphere	Poly(vinyl alcohol) (PVA)/cellulose	100–500	NIH3T3	Have the potential to be used as cell culture scaffolds.	[55]
	Poly(D,L-lactide-co-glycolide) (PLGA)	80–100	P19	Used as transplantation matrices of pluripotent stem cells for tissue engineering and regeneration.	[85]
	PLGA	50	3T3 L1 preadipocyte cells	Injectable cellular aggregates for adipose tissue engineering.	[56]
	PLGA	300–500	NIH 3T3 mouse embryo fibroblasts	Utilized as injectable and biodegradable scaffold microcarriers.	[86]
	PLGA/chitosan	200–400	Chondrocyte	As effective injectable cell carriers for cartilage tissue engineering	[40]
	Decellularized adipose tissue (DAT)	420	adipose-derived stem/stromal cells	applying dynamic culture with tissue-specific DAT microcarriers as a means of deriving regenerative cell populations.	[42]

**Table 3:** Microgels and their applications as cell carrier.

Material	Size (μm)	Cell Type	Application	Ref.
Methacrylic gelatin	100–160	Macrophage	As 3D cell culture platform.	[65]
Laminarin	100	Mouse fibroblasts cells	Support cell adhesion and expansion.	[67]
Gelatin	253	Mesenchymal stem cells	Provide a protective diffusional barrier against a pro-inflammatory environment and thereby can support the survival and differentiation of encapsulated cell.	[89]
Ploy(carboxybetaine)	10–50	hMSC, HEK-293T, and NIH-3T3	As a versatile platform for malleable cell constructs and injectable therapies.	[99]
NIPAM	200–500	Hela cell	Thermo-responsive anionic microgel scaffolds for multicellular spheroid formation.	[100]

**Table 4:** Examples of cell-laden module for constructing 3D engineering tissues.

Module type	Cell type	Assembly method	Application	Ref.
Gelatin microgel	Macrophage	Embedding into a larger tissue construct	Microtissues containing macrophage as a model cell type	[65]
PEG microgel	Human mesenchymal stem cells	Clicking the microgel blocks	Platforms for 3D cell encapsulation	[108]
PEG microgel	Dermal fibroblasts (HDF), adipose-derived mesenchymal stem cells (Ah MSC), and bone marrow-derived mesenchymal stem cells (BMh MSC)	Via a non-canonical amide linkage between the K and Q peptides mediated by activated Factor XIII	Accelerated wound healing	[109,110]
Gelatin Norbornene microgel	Bone marrow-derived mesenchymal stem cell	Covalent bonding between the microgel	Articular cartilage repair	[111]
Polyethylene glycol dimethacrylate(PEGDMA) microgel	NIH 3T3	Assembled under the drive of the applied magnetic field	Bioactive, soft 3D hydrogel constructs to be employed in soft robotics	[112]
Gelatin and collagen microspheres	Human osteoblasts, human umbilical vein endothelial cells	Randomly assembled	Engineering complex 3D tissues	[118]
Collagen gel bead	NIH 3T3 cells, Hep G2 cells, human umbilical endothelial cells (HUVECs), primary neurons, primary rat hepatocytes, and MIN6m9 cells	Stacking microtissue unit	Engineering complex 3D tissues	[113]
Gelatin-grafted-gellan microspheres	Human fetal osteoblasts, human mesenchymal stem cells	Simple mixing	Bone regeneration	[115]

## Abbreviations

2/3D	Two-/three-dimensional
ECM	Extracellular matrix
TIPS	Thermally induced phase separation
W/O	W/O
PVA	Poly(vinyl alcohol)
CNF	Cellulose nanofibril
BC	Bacterial cellulose
PEG	Poly(ethylene glycol)
U-4CR	Ugi four-component reaction
P-3CR	Passerini three-component reaction
PLA	Polylactide
PLGA	Poly(D, L-lactide-co-glycolide)
NIPAM	N-isopopylacrylamide
PEC	Polyelectrolyte complex
MSCs	Mesenchymal stem cells
PCB	Polycarboxybetaine
ZIP	Zwitterionic injectable pellet
ADSCs	Adipose-derived stem cells
CSM	Chitosan microspheres
MAPhB	Microporous annealed particle
hBMSC	Human bone marrow derived mesenchymal stem cell
HUVEC	Human umbilical vein endothelial cells
ADCs	Anchorage dependent cells

## References

[1] Asghar W., El Assal R., Shafiee H., Pitteri S., Paulmurugan R., Demirci U. (2015). Engineering cancer microenvironments for in vitro 3-D tumor models. Mater. Today.

[2] In J.G., Foulke-Abel J., Estes M.K., Zachos N.C., Kovbasnjuk O., Donowitz M. (2016). Human mini-guts: New insights into intestinal physiology and host-pathogen interactions. Nat. Rev. Gastro. Hepat..

[3] Edmondson R., Broglie J.J., Adcock A.F., Yang L. (2014). Three-dimensional cell culture systems and their applications in drug discovery and cell-based biosensors. Assay Drug Dev. Techn..

[4] Skardal A., Devarasetty M., Rodman C., Atala A., Soker S. (2015). Liver-tumor hybrid organoids for modeling tumor growth and drug response in vitro. Ann. Bio. Eng..

[5] Achilli T.-M., Meyer J., Morgan J.R. (2012). Advances in the formation, use and understanding of multi-cellular spheroids. Expert. Opin. Biol. Th..

[6] Pampaloni F., Reynaud E.G., Stelzer E.H.K. (2007). The third dimension bridges the gap between cell culture and live tissue. Nat. Rev. Mol. Cell Bio..

[7] Hollister S.J. (2005). Porous scaffold design for tissue engineering. Nat. Mater..

[8] Chen C.Y., Ke C.J., Yen K.C., Hsieh H.C., Sun J.S., Lin F.H. (2015). 3D porous calcium-alginate scaffolds cell culture system improved human osteoblast cell clusters for cell therapy. Theranostics.

[9] Mazza G., Rombouts K., Hall A.R., Urbani L., Luong T.V., Al-Akkad W., Longato L., Brown D., Maghsoudlou P., Dhillon A.P. (2015). Decellularized human liver as a natural 3D-scaffold for liver bioengineering and transplantation. Sci. Rep-UK..

[10] Song J., Chen C., Wang C., Kuang Y., Li Y., Jiang F., Gong A. (2017). Superflexible wood. ACS Appl. Mater. Inter..

[11] Khademhosseini A., Langer R., Borenstein J., Vacanti J.P. (2006). Microscale technologies for tissue engineering and biology. Proc. Natl. Acad. Sci. USA.

[12] Leong K.F., Cheah C.M., Chua C.K. (2003). Solid freeform fabrication of three-dimensional scaffolds for engineering replacement tissues and organs. Biomaterials.

[13] Loh Q.L., Choong C. (2013). Three-dimensional scaffolds for tissue engineering applications: Role of porosity and pore size. Tissue Eng. Part B-Rev..

[14] Rezwan K., Chen Q.Z., Blaker J.J., Boccaccini A.R. (2006). Biodegradable and bioactive porous polymer/inorganic composite scaffolds for bone tissue engineering. Biomaterials.

[15] Kehr N.S. (2016). Enantiomorphous periodic mesoporous organosilica-based nanocomposite hydrogel scaffolds for cell adhesion and cell enrichment. Biomacromolecules.

[16] Motealleh A., Hermes H., Jose J., Kehr N.S. (2018). Chirality-dependent cell adhesion and enrichment in Janus nanocomposite hydrogels. Nanomed-Nanotechnol..

[17] Motealleh A., Seda Kehr N. (2017). Janus nanocomposite hydrogels for chirality-dependent cell adhesion and migration. ACS Appl. Mater. Inter..

[18] Aguado B.A., Mulyasasmita W., Su J., Lampe K.J., Heilshorn S.C. (2011). Improving viability of stem cells during syringe needle flow through the design of hydrogel cell carriers. Tissue Eng. Part A.

[19] Cheng Y.H., Yang S.H., Su W.Y., Chen Y.C., Yang K.C., Cheng W.T.K., Wu S.C., Lin F.H. (2009). Thermosensitive chitosan-gelatin-glycerol phosphate hydrogels as a cell carrier for nucleus pulposus regeneration: An in vitro study. Tissue Eng. Part A.

[20] Feng Q., Wei K., Lin S., Xu Z., Sun Y., Shi P., Li G., Bian L. (2016). Mechanically resilient, injectable, and bioadhesive supramolecular gelatin hydrogels crosslinked by weak host-guest interactions assist cell infiltration and in situ tissue regeneration. Biomaterials.

[21] Wang C., Varshney R.R., Wang D.A. (2010). Therapeutic cell delivery and fate control in hydrogels and hydrogel hybrids. Adva. Drug Deliver. Rev..

[22] Lee G.Y., Kenny P.A., Lee E.H., Bissell M.J. (2007). Three-dimensional culture models of normal and malignant breast epithelial cells. Nat. Methods..

[23] Zanoni M., Pignatta S., Arienti C., Bonafè M., Tesei A. (2019). Anticancer drug discovery using multicellular tumor spheroid models. Expert Opin. Drug Dis..

[24] Sensi F., D’Angelo E., D’Aronco S., Molinaro R., Agostini M. (2019). Preclinical three-dimensional colorectal cancer model: The next generation of in vitro drug efficacy evaluation. J. Cell. Physiol..

[25] Giannattasio A., Weil S., Kloess S., Ansari N., Stelzer E.H.K., Cerwenka A., Steinle A., Koehl U., Koch J. (2015). Cytotoxicity and infiltration of human NK cells in in vivo-like tumor spheroids. BMC Cancer.

[26] Costa E.C., Moreira A.F., de Melo-Diogo D., Gaspar V.M., Carvalho M.P., Correia I.J. (2016). 3D tumor spheroids: An overview on the tools and techniques used for their analysis. Biotechnol. Adv..

[27] Valcárcel M., Arteta B., Jaureguibeitia A., Lopategi A., Martínez I., Mendoza L., Muruzabal F.J., Salado C., Vidal-Vanaclocha F. (2008). Three-dimensional growth as multicellular spheroid activates the proangiogenic phenotype of colorectal carcinoma cells via LFA-1-dependent VEGF: Implications on hepatic micrometastasis. J. Transl. Med..

[28] Roelants M., Van Cleynenbreugel B., Lerut E., Van Poppel H., de Witte P.A.M. (2011). Human serum albumin as key mediator of the differential accumulation of hypericin in normal urothelial cell spheroids versus urothelial cell carcinoma spheroids. Photoch. Photobio. Sci..

[29] Lu H., Stenzel M.H. (2018). Multicellular tumor spheroids (mcts) as a 3D in vitro evaluation tool of nanoparticles. Small.

[30] Griffith C.K., Miller C., Sainson R.C., Calvert J.W., Jeon N.L., Hughes C.C., George S.C. (2005). Diffusion limits of an in vitro thick prevascularized tissue. Tissue Eng..

[31] Nishiguchi A., Yoshida H., Matsusaki M., Akashi M. (2011). Rapid construction of three-dimensional multilayered tissues with endothelial tube networks by the cell-accumulation technique. Adv. Mater..

[32] Nishiguchi A., Matsusaki M., Asano Y., Shimoda H., Akashi M. (2014). Effects of angiogenic factors and 3D-microenvironments on vascularization within sandwich cultures. Biomaterials.

[33] Chen A.K.L., Reuveny S., Oh S.K.W. (2013). Application of human mesenchymal and pluripotent stem cell microcarrier cultures in cellular therapy: Achievements and future direction. Biotechnol. Adv..

[34] Healthcare G.E., Biosciences A. (2005). Microcarrier cell culture: Principles and methods.

[35] Van Wezel A.L. (1967). Growth of cell-strains and primary cells on micro-carriers in homogeneous culture. Nature.

[36] Abranches E., Bekman E., Henrique D., Cabral J.M. (2007). Expansion of mouse embryonic stem cells on microcarriers. Biotechnol. Bioeng..

[37] Bhuptani R.S., Patravale V.B. (2016). Porous microscaffolds for 3D culture of dental pulp mesenchymal stem cells. Int. J. Pharm..

[38] Dias A.D., Elicson J.M., Murphy W.L. (2017). Microcarriers with synthetic hydrogel surfaces for stem cell expansion. Adv. Healthc. Mater..

[39] Duan B., Zheng X., Xia Z., Fan X., Guo L., Liu J., Wang Y., Ye Q., Zhang L. (2015). Highly biocompatible nanofibrous microspheres self-assembled from chitin in NaOH/urea aqueous solution as cell carriers. Angew. Chem. Int. Edit..

[40] Fang J., Zhang Y., Yan S., Liu Z., He S., Cui L., Yin J. (2014). Poly (L-glutamic acid)/chitosan polyelectrolyte complex porous microspheres as cell microcarriers for cartilage regeneration. Acta Biomater..

[41] Melero-Martin J.M., Dowling M.A., Smith M., Al-Rubeai M. (2006). Expansion of chondroprogenitor cells on macroporous microcarriers as an alternative to conventional monolayer systems. Biomaterials.

[42] Yu C., Kornmuller A., Brown C., Hoare T., Flynn L.E. (2017). Decellularized adipose tissue microcarriers as a dynamic culture platform for human adipose-derived stem/stromal cell expansion. Biomaterials.

[43] Platen M., Mathieu E., Lück S., Schube R., Jordan R., Pauto S. (2015). Poly(2-oxazoline)-based microgel particles for neuronal cell culture. Biomacromolecules.

[44] Wang Y., Yuan X., Yu K., Meng H., Zheng Y., Peng J., Lu S., Liu X., Xie Y., Qiao K. (2018). Fabrication of nanofibrous microcarriers mimicking extracellular matrix for functional microtissue formation and cartilage regeneration. Biomaterials.

[45] Neto M.D., Oliveira M.B., Mano J.F. (2019). Microparticles in contact with cells: From carriers to multifunctional tissue modulators. Trends Biotechnol..

[46] Levato R., Visser J., Planell J.A., Engel E., Malda J., Mateos-Timoneda M.A. (2014). Biofabrication of tissue constructs by 3D bioprinting of cell-laden microcarriers. Biofabrication.

[47] Correia C.R., Bjørge I.M., Zeng J., Matsusaki M., Mano J.F. (2019). Liquefied microcapsules as dual-microcarriers for 3D+ 3D bottom-up tissue engineering. Adv. Healthcare Mater..

[48] Hernández R.M., Orive G., Murua A., Pedraz J.L. (2010). Microcapsules and microcarriers for in situ cell delivery. Adv. Drug Deliver. Rev..

[49] Tavassoli H., Alhosseini S.N., Tay A., Chan P.P., Oh S.K.W., Warkiani M.E. (2018). Large-scale production of stem cells utilizing microcarriers: A biomaterials engineering perspective from academic research to commercialized products. Biomaterials.

[50] Martin Y., Eldardiri M., Lawrence-Watt D.J., Sharpe J.R. (2011). Microcarriers and their potential in tissue regeneration. Tissue Eng. Part B Rev..

[51] Li B., Wang X., Wang Y., Gou W., Yuan X., Peng J., Guo Q., Lu S. (2015). Past, present, and future of microcarrier-based tissue engineering. J. Orthop. Transl..

[52] Chen X., Chen J., Tong X., Mei J., Chen Y., Mou X. (2020). Recent advances in the use of microcarriers for cell cultures and their ex vivo and in vivo applications. Biotechnol. Lett..

[53] Patricio T.M.F., Panseri S., Montesi M., Iafisco M., Sandri M., Tampieri A., Sprio S. (2019). Superparamagnetic hybrid microspheres affecting osteoblasts behaviour. Mat. Sci. Eng. C-Mater..

[54] Huang L., Xiao L., Poudel A.J., Li J., Zhou P., Gauthier M., Liu H., Wu Z., Yang G. (2018). Porous chitosan microspheres as microcarriers for 3D cell culture. Carbohyd. Polym..

[55] Zhang C., Zhai T., Turng L.S. (2017). Aerogel microspheres based on cellulose nanofibrils as potential cell culture scaffolds. Cellulose.

[56] Chung H.J., Park T.G. (2008). Injectable cellular aggregates prepared from biodegradable porous microspheres for adipose tissue engineering. Tissue Eng. Part A.

[57] Higashi K., Miki N. (2018). Hydrogel fiber cultivation method for forming bacterial cellulose microspheres. Micromachines.

[58] Guo M.T., Rotem A., Heyman J.A., Weitz D.A. (2012). Droplet microfluidics for high-throughput biological assays. Lab Chip.

[59] Joensson H.N., Svahn H.A. (2012). Droplet microfluidics-A tool for single-cell analysis. Angew. Chem. Int. Edit..

[60] Leng X., Zhang W., Wang C., Cui L., Yang C.J. (2010). Agarose droplet microfluidics for highly parallel and efficient single molecule emulsion PCR. Lab Chip.

[61] Mark D., Haeberle S., Roth G., Von Stetten F., Zengerle R. (2010). Microfluidic lab-on-a-chip platforms: Requirements, characteristics and applications. Microfluidics based microsystems..

[62] Kim S., Oh J., Cha C. (2016). Enhancing the biocompatibility of microfluidics-assisted fabrication of cell-laden microgels with channel geometry. Colloids Surfaces B.

[63] Aikawa T., Konno T., Takai M., Ishihara K. (2011). Spherical phospholipid polymer hydrogels for cell encapsulation prepared with a flow-focusing microfluidic channel device. Langmuir.

[64] Aikawa T., Konno T., Ishihara K. (2013). Phospholipid polymer hydrogel microsphere modulates the cell cycle profile of encapsulated cells. Soft Matter.

[65] Lee D., Lee K., Cha C. (2018). Microfluidics-Assisted Fabrication of Microtissues with Tunable Physical Properties for Developing an In Vitro Multiplex Tissue Model. Adv. Biosystems.

[66] Jiang W., Li M., Chen Z., Leong K.W. (2016). Cell-laden microfluidic microgels for tissue regeneration. Lab Chip.

[67] Martins C.R., Custódio C.A., Mano J.F. (2018). Multifunctional laminarin microparticles for cell adhesion and expansion. Carbohyd. Polym..

[68] Pajoumshariati S.R., Azizi M., Wesner D., Miller P.G., Shuler M.L., Abbaspourrad A. (2018). Microfluidic-based cell-embedded microgels using nonfluorinated oil as a model for the gastrointestinal niche. ACS Appl. Mater. Inter..

[69] Liu E.Y., Jung S., Weitz D.A., Yi H., Choi C.H. (2018). High-throughput double emulsion-based microfluidic production of hydrogel microspheres with tunable chemical functionalities toward biomolecular conjugation. Lab Chip.

[70] Hauck N., Seixas N., Centeno S., Schlüßler R., Cojoc G., Müller P., Guck J., Wöll D., Wessjohan L.A., Thiele J. (2018). Droplet-assisted microfluidic fabrication and characterization of multifunctional polysaccharide microgels formed by multicomponent reactions. Polymers.

[71] Sehlinger A., Meier M.A. (2014). Passerini and Ugi multicomponent reactions in polymer science. Multi-component and sequential reactions in polymer synthesis.

[72] Cumpstey I. (2013). Chemical modification of polysaccharides. ISRN Org. Chem..

[73] Kirschning A., Dibbert N., Dräger G. (2018). Chemical functionalization of polysaccharides-Towards biocompatible hydrogels for biomedical applications. Chem-Eur J..

[74] Heida T., Neubauer J.W., Seuss M., Hauck N., Thiele J., Fery A. (2017). Mechanically defined microgels by droplet microfluidics. Macromol. Chem. Phys..

[75] Zhang S., Ma B., Wang S., Duan J., Qiu J., Li D., Sang Y., Ge S., Liu H. (2018). Mass-production of fluorescent chitosan/graphene oxide hybrid microspheres for in vitro 3D expansion of human umbilical cord mesenchymal stem cells. Chem. Eng. J..

[76] Tan K.Y., Teo K.L., Lim J.F., Chen A.K., Reuveny S., Oh S.K. (2015). Serum-free media formulations are cell line–specific and require optimization for microcarrier culture. Cytotherapy.

[77] Guillou L., Babataheri A., Puech P.H., Barakat A.I., Husson J. (2016). Dynamic monitoring of cell mechanical properties using profile microindentation. Sci. Rep-UK..

[78] Custódio C.A., Cerqueira M.T., Marques A.P., Reis R.L., Mano J.F. (2015). Cell selective chitosan microparticles as injectable cell carriers for tissue regeneration. Biomaterials.

[79] Tedesco M.T., Di Lisa D., Massobrio P., Colistra N., Pesce M., Catelani T., Dellacasa E., Raiteri R., Martinoia S., Pastorino L. (2018). Soft chitosan microbeads scaffold for 3D functional neuronal networks. Biomaterials.

[80] Wu X.B., Peng C.H., Huang F., Kuang J., Yu S.L., Dong Y.D., Han B.S. (2011). Preparation and characterization of chitosan porous microcarriers for hepatocyte culture. Hepatob. Pancreat. Dis..

[81] Anitha A., Sowmya S., Kumar P.S., Deepthi S., Chennazhi K.P., Ehrlich H., Tsurkan M. (2014). Jayakumar, R., Chitin and chitosan in selected biomedical applications. Prog. Polym. Sci..

[82] Pellá M.G., Lima-Tenório M.K., Tenório-Neto E.T., Guilherme M.R., Muniz E.C., Rubira A.F. (2018). Chitosan-based hydrogels: From preparation to biomedical applications. Carbohyd. Polym..

[83] Hong Y., Gao C., Xie Y., Gong Y., Shen J. (2005). Collagen-coated polylactide microspheres as chondrocyte microcarriers. Biomaterials.

[84] Chen R., Curran S.J., Curran J.M., Hunt J.A. (2006). The use of poly (l-lactide) and RGD modified microspheres as cell carriers in a flow intermittency bioreactor for tissue engineering cartilage. Biomaterials.

[85] Newman K.D., McBurney M.W. (2004). Poly (D, L lactic-co-glycolic acid) microspheres as biodegradable microcarriers for pluripotent stem cells. Biomaterials.

[86] Kim T.K., Yoon J.J., Lee D.S., Park T.G. (2006). Gas foamed open porous biodegradable polymeric microspheres. Biomaterials.

[87] Zhao X., Liu S., Yildirimer L., Zhao H., Ding R., Wang H., Cui W., Weitz D. (2016). Injectable stem cell-laden photocrosslinkable microspheres fabricated using microfluidics for rapid generation of osteogenic tissue constructs. Adv. Funct. Mater..

[88] Li F., Truong V.X., Thissen H., Frith J.E., Forsythe J.S. (2017). Microfluidic encapsulation of human mesenchymal stem cells for articular cartilage tissue regeneration. ACS Appl. Mater. Inter..

[89] Sung B., Krieger J., Yu B., Kim M.H. (2018). Colloidal gelatin microgels with tunable elasticity support the viability and differentiation of mesenchymal stem cells under pro-inflammatory conditions. J. Biomed Mater. Res. A.

[90] Gjorevski N., Sachs N., Manfrin A., Giger S., Bragina M.E., Ordóñez-Morán P., Clevers H., Lutolf M.P. (2016). Designer matrices for intestinal stem cell and organoid culture. Nature.

[91] Laschewsky A. (2014). Structures and synthesis of zwitterionic polymers. Polymers.

[92] Jiang S., Cao Z. (2010). Ultralow-fouling, functionalizable, and hydrolyzable zwitterionic materials and their derivatives for biological applications. Adv. Mater..

[93] Shao Q., Jiang S. (2015). Molecular understanding and design of zwitterionic materials. Adv. Mater..

[94] Ishihara K., Goto Y., Takai M., Matsuno R., Inoue Y., Konno T. (2011). Novel polymer biomaterials and interfaces inspired from cell membrane functions. BBA-Gen. Subj..

[95] White A.D., Nowinski A.K., Huang W., Keefe A.J., Sun F., Jiang S. (2012). Decoding nonspecific interactions from nature. Chem. Sci..

[96] Bai T., Sun F., Zhang L., Sinclair A., Liu S., Ella-Menye J.R., Zheng Y., Jiang S. (2014). Restraint of the differentiation of mesenchymal stem cells by a nonfouling zwitterionic hydrogel. Angew. Chem. Int. Edit..

[97] Li B., Yuan Z., Zhang P., Sinclair A., Jain P., Wu K., Tsao C., Xie J., Bai T., Jiang S. (2018). Zwitterionic nanocages overcome the efficacy loss of biologic drugs. Adv. Mater..

[98] Zhang P., Sun F., Tsao C., Liu S., Jain P., Sinclair A., Hung H.C., Bai T., Wu K., Jiang S. (2015). Zwitterionic gel encapsulation promotes protein stability, enhances pharmacokinetics, and reduces immunogenicity. P Natl. Acad. Sci..

[99] Sinclair A., O’Kelly M.B., Bai T., Hung H.C., Jain P., Jiang S. (2018). Self-Healing Zwitterionic Microgels as a Versatile Platform for Malleable Cell Constructs and Injectable Therapies. Adv. Mater..

[100] Cui X., Hartanto Y., Wu C., Bi J., Dai S., Zhang H. (2018). Tuning microenvironment for multicellular spheroid formation in thermo-responsive anionic microgel scaffolds. J. Biomed. Mater. Res. A.

[101] Tripathi A., Melo J.S. (2015). Preparation of a sponge-like biocomposite agarose–chitosan scaffold with primary hepatocytes for establishing an in vitro 3D liver tissue model. RSC Adv..

[102] Yan S., Wei J., Liu Y., Zhang H., Chen J., Li X. (2015). Hepatocyte spheroid culture on fibrous scaffolds with grafted functional ligands as an in vitro model for predicting drug metabolism and hepatotoxicity. Acta Biomater..

[103] Yan S., Xia P., Xu S., Zhang K., Li G., Cui L., Yin J. (2018). Nanocomposite porous microcarriers based on strontium-substituted HA-g-poly (γ-benzyl-l-glutamate) for bone tissue engineering. ACS Appl. Mater. Inter..

[104] Choi S.W., Xie J., Xia Y. (2009). Chitosan-based inverse opals: Three-dimensional scaffolds with uniform pore structures for cell culture. Adv. Mater..

[105] Murphy C.M., Haugh M.G., O’Brien F.J. (2010). The effect of mean pore size on cell attachment, proliferation and migration in collagen–glycosaminoglycan scaffolds for bone tissue engineering. Biomaterials.

[106] O’Brien F.J., Harley B.A., Yannas I.V., Gibson L.J. (2005). The effect of pore size on cell adhesion in collagen-GAG scaffolds. Biomaterials.

[107] Yannas I.V., Lee E., Orgill D.P., Skrabut E.M., Murphy G.F. (1989). Synthesis and characterization of a model extracellular matrix that induces partial regeneration of adult mammalian skin. P. Natl. Acad. Sci..

[108] Caldwell A.S., Campbell G.T., Shekiro K.M., Anseth K.S. (2017). Clickable microgel scaffolds as platforms for 3D cell encapsulation. Adv. Healthc Mater..

[109] Griffin D.R., Weaver W.M., Scumpia P.O., Di Carlo D., Segura T. (2015). Accelerated wound healing by injectable microporous gel scaffolds assembled from annealed building blocks. Nat. Mater..

[110] Nih L.R., Sideris E., Carmichael S.T., Segura T. (2017). Injection of microporous annealing particle (MAP) hydrogels in the stroke cavity reduces gliosis and inflammation and promotes NPC migration to the lesion. Adv. Mater..

[111] Li F., Truong V.X., Fisch P., Levinson C., Glattauer V., Zenobi-Wong M., Thissen H., Forsythe J.S., Frith J.E. (2018). Cartilage tissue formation through assembly of microgels containing mesenchymal stem cells. Acta Biomater..

[112] Tasoglu S., Yu C.H., Gungordu H.I., Guven S., Vural T., Demirci U. (2015). Guided and magnetic self-assembly of tunable magnetoceptive gels. Nat. Commun..

[113] Matsunaga Y.T., Morimoto Y., Takeuchi S. (2011). Molding cell beads for rapid construction of macroscopic 3D tissue architecture. Adv. Mater..

[114] McGuigan A.P., Leung B., Sefton M.V. (2006). Fabrication of cells containing gel modules to assemble modular tissue-engineered constructs. Nat. Protoc..

[115] Wang C., Gong Y., Zhong Y., Yao Y., Su K., Wang D.A. (2009). The control of anchorage-dependent cell behavior within a hydrogel/microcarrier system in an osteogenic model. Biomaterials.

[116] Son J., Bae C.Y., Park J.K. (2015). Freestanding stacked mesh-like hydrogel sheets enable the creation of complex macroscale cellular scaffolds. Biotechnol. J..

[117] Tiruvannamalai-Annamalai R., Armant D.R., Matthew H.W. (2014). A glycosaminoglycan based, modular tissue scaffold system for rapid assembly of perfusable, high cell density, engineered tissues. PLoS ONE.

[118] Zhong M., Wei D., Yang Y., Sun J., Chen X., Guo L., Wei Q., Wan Y., Fan H., Zhang X. (2017). Vascularization in engineered tissue construct by assembly of cellular patterned micro-modules and degradable microspheres. ACS Appl. Mater. Inter..

